# Congenital Myasthenic Syndrome in a Mixed Breed Dog

**DOI:** 10.3389/fvets.2017.00173

**Published:** 2017-10-17

**Authors:** Theresa J. Blakey, Jennifer R. Michaels, Ling T. Guo, Amy J. Hodshon, G. Diane Shelton

**Affiliations:** ^1^Department of Small Animal Clinical Sciences, University of Tennessee College of Veterinary Medicine, Knoxville, TN, United States; ^2^Department of Pathology, Comparative Neuromuscular Laboratory, School of Medicine, University of California San Diego, LaJolla, CA, United States

**Keywords:** congenital myasthenic syndrome, myasthenia gravis, dog, pyridostigmine bromide, mixed breed, *CHRNE*

## Abstract

A 6-month-old, male, intact mixed breed dog was presented for a 3-month history of progressive generalized weakness. Neurologic examination revealed non-ambulatory tetraparesis, weakness of the head and neck, and decreased withdrawal reflexes in all limbs consistent with a generalized neuromuscular disorder. Electromyography and motor nerve conduction velocity were normal. Repetitive nerve stimulation showed a decremental response of the compound muscle action potential with improvement upon intravenous administration of edrophonium chloride. The serum acetylcholine receptor (AChR) antibody titer was within reference range. Cerebrospinal fluid analysis was unremarkable. A presumptive diagnosis of post-synaptic congenital myasthenic syndrome (CMS) was made. Treatment with pyridostigmine bromide was initiated with titrated increases in dosage resulting in an incomplete improvement in clinical signs. The dog was euthanized 2 months after initiation of treatment due to poor quality of life. Immunostaining for localization of antibodies against end-plate proteins in muscle biopsies was negative. Immunofluorescence staining for AChRs in external intercostal muscle biopsies showed absence of AChRs and biochemical quantitation showed a markedly decreased concentration of AChRs with no detectable AChR-bound autoantibody which confirmed the diagnosis of a CMS. Evaluation for the *CHRNE* mutation previously identified as the causative mutation of CMS in Jack Russell Terriers was performed and was negative. This is the first reported confirmed case of CMS in a mixed breed dog and provides a review of typical clinical and diagnostic findings as well as treatment considerations.

## Case Presentation

A 6-month-old, male, intact mixed breed dog [6.54 kg (14.4 lb)] was referred with a 3-month history of progressive generalized weakness. The dog was adopted at approximately 3 months of age after having been found as a stray. At the time of adoption, the dog was perceived to be normal. About 1 week later, it was noted that the dog developed an abnormal, stiff gait after playing and would sometimes fall over. The dog was also having difficulty posturing to defecate. Despite empirical treatment with clindamycin (12.3 mg/kg, PO, BID) and steroids (unknown type or dose), the dog displayed progressive episodes of weakness and falling following activity that eventually progressed to non-ambulatory tetraparesis over the next several months.

On presentation, physical examination revealed grade 3/6 left and right apical systolic murmurs. Echocardiogram revealed mitral valve dysplasia causing systolic anterior motion of the mitral valve with severe dynamic left ventricular outflow tract obstruction and mild mitral regurgitation. In addition, there was a mild dynamic right ventricular outflow tract obstruction. Atenolol (1 mg/kg, PO, BID) was initiated. On neurologic examination, the dog was non-ambulatory tetraparetic (lower motor neuron quality with poor muscle tone) and could only lift the head off of the floor for a few seconds at a time. Postural reactions were decreased in all limbs; however, they were stronger than expected given the profound degree of weakness. Cranial nerve examination was normal including palpebral reflex. Withdrawal reflexes were reduced in all limbs. Patellar reflexes were normal in both pelvic limbs. Both the palpebral and patellar reflexes were normal even with repeated stimulation. Findings were consistent with a diffuse neuromuscular disease.

**Figure 1:**
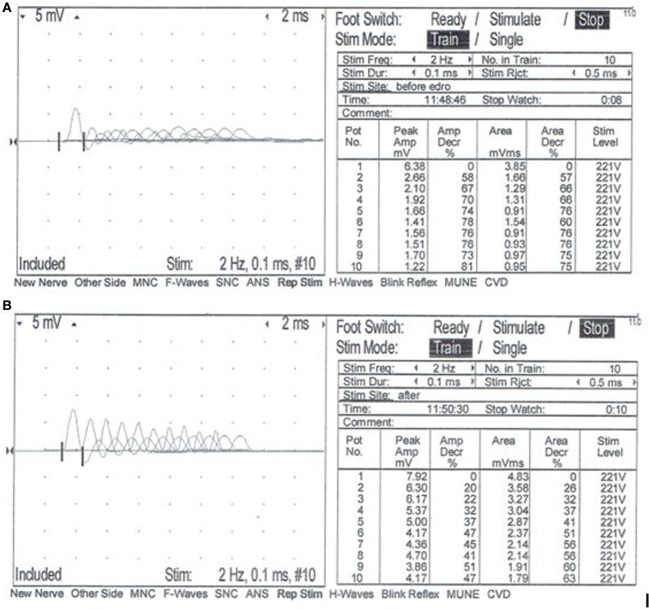
Repetitive nerve stimulation at the time of initial evaluation. Repetitive stimulation of the tibial nerve performed before **(A)** and after **(B)** intravenous administration of edrophonium chloride (0.1 mg/kg). Nerve stimulation was performed at the level of the hock, and complex muscle action potentials (CMAP) were recorded from the interosseous muscle. Stimulus rate = 2 Hz. Stimulus duration = 0.1 ms. Repetitions = 10. Normal response = no decrement >10% of the starting CMAP amplitude or area. **(A)** Repetitive nerve stimulation prior to administration of edrophonium chloride showed a decrement of 74% between the first and fifth waves. **(B)** Repetitive nerve stimulation after administration of edrophonium chloride showed a persistent but less severe decrement of 37% between the first and fifth waves.

A complete blood count, serum biochemistry panel, and urinalysis were performed. All values were within normal limits, including serum creatine kinase activity. Electromyographic (EMG) evaluation of the thoracic and pelvic limbs showed no spontaneous activity. Motor nerve conduction studies of the ulnar nerve (with stimulation at the elbow and carpus) and sciatic/tibial nerves (with stimulation at the hip and hock) were performed; M-waves were recorded from the interosseous muscle of the thoracic limb and pelvic limb, respectively. M-wave morphology and motor nerve conduction velocities of the ulnar and sciatic/tibial nerves were within normal limits at 70 and 68 m/s, respectively. Repetitive stimulation of the tibial nerve at the level of the hock with a stimulus rate of 2 Hz revealed a decrement in compound muscle action potential (CMAP) amplitude of 74% between the first and fifth waves (Figure 1A). Repetitive nerve stimulation following administration of edrophonium (0.1 mg/kg, IV) using the same stimulus parameters showed a persistent but improved decrement in CMAP amplitude of 37% between the first and fifth waves (Figure 1B). A cisternal spinal tap was performed; cerebrospinal fluid analysis was normal. These diagnostic findings were most consistent with a disorder of neuromuscular transmission. Based on the concern for myasthenia gravis (MG), thoracic radiographs were performed and were normal. Following recovery from general anesthesia, an edrophonium chloride challenge test was performed (0.1 mg/kg, IV). This resulted in partial improvement of the dog’s generalized muscle weakness in that it was then able to lift and move the head normally, stand in a crouched position, and take a few steps unsupported (Videos S1 and S2 in Supplementary Material). An acetylcholine receptor (AChR) antibody test was performed and was within normal limits [0.01 nmol/L (reference ≤0.6 nmol/L)] making acquired AChR associated MG highly unlikely. Following the diagnostic evaluation, the top differential was a congenital myasthenic syndrome (CMS). The dog was started on pyridostigmine bromide (0.5 mg/kg, PO, BID).

After initiation of pyridostigmine bromide therapy, the dog showed improvement in the ability to lift the head, rise into a crouched standing position, and take a few steps before falling. Over the next 2 months, the pyridostigmine bromide dosage and frequency were increased every 5–10 days, but the clinical signs did not improve. At the maximal dosage administered (1.4 mg/kg, PO, TID), the dog began showing signs of cholinergic overdose including excessive lacrimation and worsening weakness. After 2 months of attempted treatment, the dog was euthanized due to lack of continued improvement with treatment and poor quality of life.

**Figure 2:**
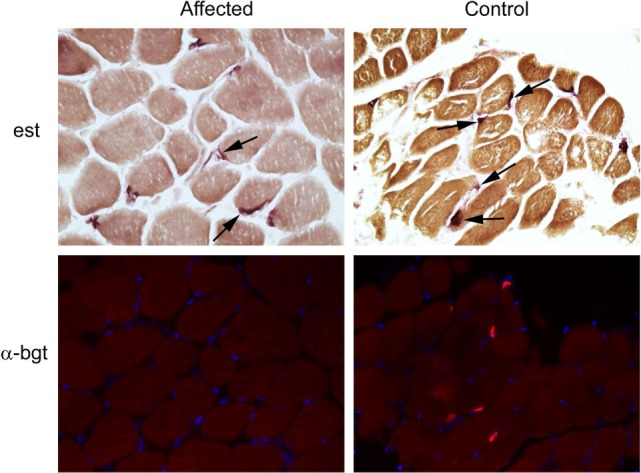
Localization of motor end-plates and acetylcholine receptors (AChRs) in external intercostal muscle of a dog with congenital myasthenic syndrome (CMS). End-plate AChRs were non-detectable using immunofluorescent staining of cryosections of the external intercostal muscle in the affected CMS dog and stained appropriately in control sections. Cryosections from the affected dog and control archived tissue were reacted for esterase to identify neuromuscular junctions. Serial sections were reacted with labeled α-bungarotoxin (α-bgt) for identification of AChRs (highlighted in red). End-plates are noted with arrows in both the affected and control muscles. End-plates are present in the muscle from the CMS dog but AChRs are not found.

Following euthanasia, external intercostal muscle (collected origin to insertion) and cranial tibial muscle biopsies were collected, immediately frozen, and stored at −80°C until shipped on dry ice to the Comparative Neuromuscular Laboratory, University of California San Diego. Muscle AChR concentration and antibody bound AChR were biochemically quantitated in the external intercostal muscle by previously published methods (1). The AChR concentration was 0.1 pmol/g tissue (reference 0.2–0.4 pmol/g tissue) with no detectable antibody bound to AChRs. This finding supported skeletal muscle AChR deficiency consistent with a CMS. Histochemical and cytochemical staining of muscle cryosections (8 µm) was performed for localization of motor end-plates using the esterase reaction with serial sections stained for localization of AChRs using labeled α-bungarotoxin by immunofluorescence (2). Compared to archived control muscle, motor end-plates were identified but AChRs were undetectable (Figure 2) further supporting a diagnosis of CMS.

Additional muscle (biceps femoris) and peripheral nerve (fibular) samples were shipped unfixed and chilled or immersion fixed in 10% neutral buffered formalin to the Comparative Neuromuscular Laboratory. Other myopathies and neuropathies were ruled out histologically by standard methodologies. Furthermore, antibodies against end-plate proteins in general were not detected using the immunoreagent staphylococcal protein A-horseradish peroxidase making a diagnosis of other causes of AChR seronegative acquired (immune-mediated) MG unlikely.

Best veterinary care was practiced in the clinical and diagnostic evaluation as well as treatments. The owner of the dog provided informed consent for all procedures prior to them being performed. As no experimental protocols were utilized, an institutional review was not required or performed.

## Background

Acquired MG is the most common neuromuscular disorder described in dogs. It has been reported in a variety of pure breeds and mixed breed dogs with a predisposition in the Newfoundland, Akita, Chihuahua, German Short-Haired Pointer, and terrier breeds (1, 3). Circulating autoantibodies against AChRs at the nicotinic neuromuscular junction result in the loss of AChRs and impaired neuromuscular transmission.

Unlike autoimmune MG, CMSs are rare and not associated with circulating AChR antibodies; rather, they are caused by inherited defects in genes involved in the development or maintenance of various pre-synaptic, synaptic, or post-synaptic proteins critical to neuromuscular transmission (4). Causative defects include abnormalities of the synaptic vesicles containing acetylcholine (ACh), the formation of ACh by choline acetyltransferase, or AChR (4). In people, over 20 genetic mutations that cause CMS have been identified (4). Post-synaptic CMS, specifically defects in the AChR, are most commonly reported in people and account for 51% of CMS cases (4). Causative mutations have been identified in a few cases of CMS in dogs and cats.

## Discussion

Congenital myasthenic syndromes have been reported in Smooth Fox Terriers, Springer Spaniels, Long-haired Miniature Dachshunds, Jack Russell Terriers, Labrador Retrievers, Golden Retrievers, and Old Danish Pointing dogs (Gammel Dansk Hønsehund) (1, 2, 5–9). There is a single report of two mongrel dogs with suspected CMS; however, confirmatory diagnostic testing was not performed (10). CMS has also been reported in Sphynx and Devon Rex cats as well as Brahman calves (11–13). To the authors’ knowledge, this is the first report describing a confirmed case of CMS in a mixed breed animal.

As stated above, there are over 20 genetic mutations associated with CMS in people with over half of cases caused by a defect in AChR subunits (4). The adult AChR is composed of five subunits each coded by a specific gene, 2 alpha (α; *CHRNA1*), 1 beta (β; *CHRNB1*), 1 delta (δ; *CHRND*), and 1 epsilon (ε; *CHRNE*). The ε subunit replaces the fetal gamma (γ; *CHRNG*) subunit to form the adult AChR (4). The majority of human CMS cases are due to a post-synaptic dysfunction of neuromuscular transmission, the most common cause of which is a primary AChR deficiency (4). Mutations of the *CHNRE* gene are responsible for almost all primary AChR deficiencies in people (4). The mutated ε subunit is replaced with the fetal γ subunit allowing for some expression of the AChR complement; however, this expression is typically less than 10% of normal (4). While mutations in the *CHRNA, CHRNB*, and *CHRND* genes do occur, they are almost uniformly associated with fetal death due to the lack of substitution subunits (4).

The majority of reported CMS cases in animals is suspected or proven to be due to a primary AChR deficiency. This has been well established in the Smooth Fox Terrier, Springer Spaniel, Long-haired Miniature Dachshund, and Jack Russell Terrier breeds (1, 2, 7, 9). A frameshift mutation in *CHRNE* has been confirmed in Jack Russell Terriers with CMS (3, 5). A *CHRNE* mutation was also identified as the cause of CMS in Brahman calves (13). CMS in Labrador Retrievers and Sphynx and Devon Rex cats is the result of a mutation in the *COLQ* gene, which encodes the collagenous tail of acetylcholinesterase (AChE) (5, 6, 8, 11, 12). In the Old Danish Pointing dog breed, however, CMS is associated with pre-synaptic dysfunction of neuromuscular transmission and a mutation in the choline acetyl-transferase (*CHAT*) gene (6). This gene codes for choline acetyltransferase, which is responsible for formation of ACh in the nerve terminal, the lack of which results in a pre-synaptic CMS (6).

Ante-mortem diagnosis of CMS can be challenging. Clinical signs are often indistinguishable from MG and many other congenital myopathies and neuropathies. Progressive, generalized skeletal muscle fatigue characteristically propagated and worsened by persistent exercise is the most common clinical sign affecting dogs with CMS (3). There are some notable differences between clinical signs in CMS versus MG. It has been reported that up to 43% of dogs with MG have focal forms affecting specific muscles or muscle groups such as the esophagus or extraocular muscles (9, 14), whereas CMS reportedly causes almost exclusively generalized signs. Regurgitation secondary to megaesophagus is a common clinical feature of MG, affecting up to 90% of dogs (9), but megaesophagus has only been described in the Smooth Fox Terrier breed with CMS (7). The dog described in this report was not affected by megaesophagus, nor was regurgitation observed.

Because of the overlapping clinical features of CMS and other causes of neuromuscular weakness, signalment is critical to identification of CMS as a primary differential diagnosis. While multiple reports of CMS in a limited number of pure breeds might have discouraged the consideration of CMS in atypical breeds, this report demonstrates the need to include CMS as a differential diagnosis for all breeds, including mixed breed dogs. Although the ancestry of mixed breed dogs is often unknown (as is the case with the dog in this report), these animals may have familial association with known affected breeds or be inbred resulting in manifestation of rare genetic disease such as CMS. CMS manifests in young puppies with onset of clinical signs typically occurring between the time of weaning and when the pups begin to ambulate. Most cases are presented between 6 and 12 weeks of age (1, 3). MG almost exclusively occurs in adult dogs with only 1 report of acquired AChR seropositive MG in a 7-week-old puppy (15) Generalized weakness in the dog in this report was not noted until approximately 3.5 months of age. However, it is possible that earlier weakness may have gone undocumented as the dog was a stray prior to adoption.

A classic electrophysiologic finding associated with MG and other forms of post-synaptic neuromuscular transmission dysfunction is a decrement in the CMAP during repetitive nerve stimulation (9). Although this finding is suggestive of CMS and MG, it is not specific for these diseases. Other diseases such as botulism, myositis, and motor neuropathies can cause a similar decremental response. A decremental response following repetitive nerve stimulation coupled with normal EMG, motor nerve conduction velocities and relatively normal muscle and nerve biopsies further support a diagnosis of CMS or MG.

The therapeutic response to an AChE inhibitor such as edrophonium or pyridostigmine can aide in reaching the diagnosis of CMS. Cases of CMS associated with reduced or non-detectable AChR at the motor end-plates can demonstrate transient or partial improvement in clinical signs following administration of intravenous edrophonium or oral pyridostigmine. However, in cases of pre-synaptic or synaptic CMS, administration of AChE inhibitors may not lead to clinical improvement or can even cause clinical signs to worsen such as occurs in CMS associated with mutations in *COLQ* (2, 4). This difference in response to AChE inhibitors is frequently used in the initial evaluation of human CMS patients in order to categorize the location of the defect (4). A similar lack of improvement or worsening in cases of pre-synaptic or synaptic CMS has been demonstrated in the Labrador Retriever and Old Danish Pointing dog breeds (2, 6, 8). The dog in this report had a positive electrophysiological and clinical response to AChE inhibitors, which supports a post-synaptic CMS defect; this was confirmed post-mortem.

Definitive ante-mortem diagnosis of CMS in dogs is challenging. In particular, distinguishing between CMS and seronegative MG can be especially difficult. While seronegative MG was a differential for the dog in this report, there were several factors that contributed to the ultimate diagnosis of CMS. As described above, the dog’s signalment, clinical signs, electrodiagnostic test results, and therapeutic response were most consistent with CMS. The absence of staining of motor end-plates for bound antibodies in muscle biopsies using the immunoreagent SPA-HRPO ruled out the presence of antibodies against any end-plate proteins that could result in seronegative MG.

While serologic testing for AChR autoantibodies is the “gold standard” for diagnosis of acquired AChR related MG, about 2% of generalized MG cases will be seronegative (9). The dog in this report was seronegative making a diagnosis of MG unlikely but still possible. The biochemical quantification of AChRs in the external intercostal muscle documented the low AChR content and the absence of AChR antibodies bound in muscle, thus, ruled out seronegative AChR-related MG caused by low-titer high-affinity antibodies. The absence of staining with the immunoreagent SPA-HRPO in the muscle biopsies ruled out other forms of seronegative MG. These findings confirmed a CMS associated with low AChR content and ruled out acquired causes of autoimmune MG.

Ultimately, identification of a causative mutation is the gold standard for diagnosis of a CMS. Genetic testing for known mutations is available, and as additional CMS-causing mutations are identified, genetic testing may provide a minimally invasive, definitive ante-mortem diagnostic option. In addition, the ability to perform full genome sequencing in veterinary medicine provides another avenue to evaluate genes responsible for CMS thereby potentially allowing us to discover previously unknown genetic mutations responsible for disease in animals. The dog in this report was only evaluated for the Jack Russell Terrier *CHRNE* gene mutation as the history, electrodiagnostic findings, and response to therapy were most consistent with a post-synaptic dysfunction of neuromuscular transmission similar to that described in Jack Russell Terriers (2). The dog was negative for this specific mutation; however, this does not rule out the possibility of a different mutation affecting *CHRNE*. Further genetic screening was not performed.

Therapy for CMS is limited, and the outcome in most dogs with CMS is poor. Unlike MG, CMS is not associated with autoantibodies affecting AChR; therefore the use of immunosuppression is unwarranted and unsuccessful, and treatment of post-synaptic CMS with end-plate AChR deficiency is limited to AChE inhibition. AChE inhibitor therapy can be transiently successful in controlling clinical signs in post-synaptic CMS; however, drug resistance can develop. Many dogs, including the dog in this report, exhibit progressive clinical signs despite increasing dosages of anticholinesterase drugs.

Additional treatments in people with CMS depend on the form of the disease. With primary AChR deficiency, a combination of pyridostigmine and amifampridine (3,4-diaminopyridine) is recommended. Amifampridine increases the number of ACh quanta released at the nerve terminal by each nerve impulse. In combination, these two treatments can improve neuromuscular transmission. Albuterol is a selective β2-adrenergic agonist and has been recommended in the treatment of AChE deficiency and other myasthenic syndromes (4). The mechanism by which albuterol improves neuromuscular transmission is not understood (4). Treatment options with other forms of CMS may include quinidine, fluoxetine, and/or ephedrine (4). Additional treatment other than pyridostigmine was not attempted in this dog.

Long-term survival in dogs with CMS is difficult to assess as the subject has been poorly evaluated and documented, but appears to be variably poor. Most dogs do not survive past 1 year of age. The exception is the Smooth-Haired Miniature Dachshund breed, which can undergo spontaneous remission with resolution of clinical signs by 6 months of age (1). An explanation for this remission is not known but may involve delayed maturation in the switch from fetal to adult AChR subunits.

## Concluding Remarks

In conclusion, CMS should be considered a rare differential diagnosis for all dogs with generalized weakness starting at or before 6–12 weeks of age irrespective of breed. This report describes classic clinical and diagnostic features of CMS in an atypical breed. The poor outcome despite treatment is consistent with the existing veterinary literature regarding CMS.

## Ethics Statement

Best veterinary care was practiced in the clinical and diagnostic evaluation as well as treatments. The owner of the dog provided informed consent for all procedures prior to them being performed. As no experimental protocols were utilized, an institutional review was not required or performed.

## Author Contributions

TB: initial manuscript generation and minor manuscript editing. JM: patient assessment, diagnostic testing and interpretation, and treatment; assisted with initial manuscript generation; major manuscript editing; and manuscript submission. LG: biopsy sample testing and interpretation. AH: supervision of patient assessment, diagnostic testing and interpretation, and treatment; and manuscript editing. GS: biopsy sample testing and interpretation; and manuscript editing.

## Conflict of Interest Statement

The authors declare that the research was conducted in the absence of any commercial or financial relationships that could be construed as a potential conflict of interest.

## Supplementary Material

Video S1 Video of the dog at initial presentation. The dog was non-ambulatory tetraparetic (lower motor neuron quality) and could only lift the head off of the floor for a few seconds at a time.

Video S2 Video of the dog at initial presentation, following administration of edrophonium chloride (0.1 mg/kg, IV). This resulted in partial improvement of the dog’s generalized muscle weakness. The dog was able to lift and move the head normally, stand in a crouched position, and take a few steps unsupported.
